# Evaluation of Factors Associated with Pulmonary Complications in Patients Undergoing Surgery for Epithelial Ovarian Cancer

**DOI:** 10.3390/jcm14041314

**Published:** 2025-02-16

**Authors:** Aysun Alci, Necim Yalcin, Mustafa Gokkaya, Gulsum Ekin Sari, Harun Turkmenoglu, Ulku Arslan, Isin Ureyen, Tayfun Toptas

**Affiliations:** 1 Department of Gynecologic Oncology, Antalya Training and Research Hospital, Health Sciences University, 07100 Antalya, Turkey; aysun_alci@hotmail.com (A.A.); zinaryal@gmail.com (N.Y.); mugokkaya@gmail.com (M.G.); drekingulsum@gmail.com (G.E.S.); harunturkmenoglu01@gmail.com (H.T.); isin.ureyen@gmail.com (I.U.); drttoptas@gmail.com (T.T.); 2 Department of Anesthesiology and Reanimation, Faculty of Medicine, Akdeniz University, 07100 Antalya, Turkey

**Keywords:** cytoreductive surgery, pulmonary complications, ovarian cancer

## Abstract

**Background:** Ovarian cancer surgery requires multiple radical resections with a high risk of complications. The objective of this single-centre, retrospective study was to identify the factors associated with pulmonary complications following cytoreduction. **Methods:** The study included 179 patients who underwent surgery at the gynaecological oncology department of the Antalya Training and Research Hospital between January 2015 and December 2021. A univariate analysis was performed to identify significant risk factors for postoperative pulmonary complications. The data obtained were then subjected to multivariate analysis to determine the relative importance of each factor. **Results:** A total of 176 ovarian cancer patients underwent cytoreductive surgical procedures for epithelial ovarian cancer (EOC) during the study period. Postoperative pulmonary complications (PPCs) occurred in a total of 24 patients (13.4%). Of the complications observed, *n* = 18 (10.06%) were pulmonary effusion, *n* = 5 (2.79%) were pulmonary thromboembolism, *n* = 1 (0.56%) was pneumo-mediastinum, *n* = 6 (3.35%) were pulmonary oedema, and *n* = 1 (0.56%) was transfusion-related lung injury (TRALI). Pulmonary complication rates were 6.512 times higher in patients who underwent diaphragm peritonectomy (*p* = 0.014) and 26.1 times higher in smokers (*p* = 0.005). When an ROC analysis was performed for quantitative parameters related to pulmonary complications, the sensitivity and specificity of the duration of surgery were 83.3% and 64.5%, respectively, and the sensitivity and specificity of the duration of postoperative hospital stay were 79.2% and 67.5% (*p* < 0.001, *p* < 0.001, *p* < 0.001, *p* < 0.001). **Conclusions:** An improved understanding of the multifactorial aetiology of PPCs and the development of an appropriate perioperative management strategy may serve to mitigate the negative impact of these complications, thereby contributing to an enhancement in patient outcomes.

## 1. Introduction

Ovarian cancer is the most common gynaecological malignancy with a poor prognosis [1]. The primary treatment approach is platinum-based combination chemotherapy after debulking surgery to remove all visible disease or at least reduce the diameter of tumour implants to less than 1 cm [2]. The majority of disease is diagnosed at stage III, at which point upper abdominal surgery is required. Extensive upper abdominal surgery, including diaphragmatic peritonectomy, splenectomy, and distal pancreatectomy, may improve survival by reducing the rate of residual disease in patients with advanced EOC [3]. However, it may also increase the incidence of postoperative complications [4]. In a study of 320 patients who underwent surgery for epithelial ovarian cancer, *n* = 30 (44.8%) had mild complications, *n* = 7 (10.4%) had severe complications, and the 90-day mortality was *n* = 2 (2.98%) [5].

Postoperative complications are still unavoidable despite rapid improvements in perioperative management and surgical techniques. Postoperative complications can have a negative impact on survival by delaying the time to start adjuvant therapy after surgery [6]. Several reports have shown that pleural effusion is the most common pulmonary complication following debulking surgery [7,8]. The development of postoperative complications is influenced by a number of factors, including age, comorbidities, and modifiable risk factors such as smoking and mobilisation. Additionally, surgical and anaesthetic considerations, such as operative time, surgical radicality, operation position, blood and blood product transfusions, and IV fluid resuscitation can also affect the risk of postoperative complications [9,10]. Postoperative pulmonary complications (PPCs) represent the most prevalent complication affecting the respiratory system subsequent to anaesthesia and surgery [11]. The risk factors for the development of PPCs are intricate, and it is imperative for clinicians to identify these patients and optimise risk factors to enhance standards of care [12]. The development of mild pulmonary complications has been demonstrated to increase the early postoperative mortality, the probability of ICU admission, and the duration of hospital stay in patients [13]. This has the potential to increase mortality and morbidity rates, as well as the economic burden on health systems. A paucity of studies exists in the extant literature that analyse the incidence of pulmonary complications and related factors in cytoreductive surgery [11]. A more profound comprehension of this issue is of paramount importance in terms of averting pulmonary complications and related increased mobility/mortality in this patient group, as well as mitigating the economic burden on healthcare systems due to prolonged hospitalisation and intensive care unit admission (ICU) due to complications.

The aim of this study was to identify factors associated with PPCs in patients undergoing primary or interval cytoreductive surgery for EOC. It is conceivable that some of these risk factors can be modified preoperatively by healthcare professionals to improve future postoperative outcomes. Furthermore, the use of early diagnostic methods in patients with a high potential for developing PPCs may facilitate a more accurate understanding of the situation.

## 2. Methods

The current study was conducted in accordance with the Declaration of Helsinki and approved by the Ethics Committee of the Antalya Training and Research Hospital, Antalya, Turkey (Approval number 6/26, date 9 May 2024). Patient information was obtained by a retrospective analysis of daily medical records and patient files. This was a retrospective study. Informed consent was not obtained. The study included 179 patients over 18 years of age who underwent cytoreductive surgery for ovarian cancer in the Gynaecological Oncology Department of the Antalya Training and Research Hospital between January 2015 and December 2021. Patients under 18 years of age and patients operated on for other gynaecological malignancies were excluded from the study. The most widely accepted definition of PPCs in the literature is “Conditions affecting the respiratory tract that may adversely affect the clinical course of the patient after surgery”, and there is a wide spectrum of pulmonary complications, including atelectasis, pneumonia, bronchitis, bronchospasm, lung collapse due to airway mucus obstruction, respiratory failure with mechanical ventilation for >48 h, aspiration pneumonia, pneumomediastinum, pulmonary oedema, pulmonary effusion, transfusion-related acute lung injury, and acute respiratory distress syndrome. In our study, we included the complications that required the least amount of pharmacological treatment [14,15,16]. The clinicopathological characteristics of the patients included age, Eastern Cooperative Oncology Group (ECOG) performance status, cardiac and pulmonary comorbidities, the presence of diabetes mellitus (DM), and smoking history. Patients with major cardiac comorbidities included atherosclerotic disease, congestive heart failure, valvular disease, and arrhythmias, while major pulmonary comorbidities included asthma and chronic obstructive pulmonary disease. Pleural pulmonary nodules, paracardiac lymph nodes, and pleural ascites associated with preoperative ovarian cancer were recorded from preoperative imaging. Intraoperative observation was recorded for the presence of diaphragmatic disease, the presence and volume of ascites, interval or primary debulking surgery according to the need for neoadjuvant chemotherapy according to current literature [17,18], and the type of cytoreduction performed (maximal, optimal, suboptimal). Surgical resections (peritonectomy, diaphragmatic peritonectomy, splenectomy/pancreatectomy, lymphadenectomy) and disease FIGO 2014 stage [19] and tumour histotype according to surgical staging at the end of resections were recorded. The following variables were recorded: presence of intraoperative complications, need for blood transfusion, operation time, preoperative Ca125 and albumin levels, albumin level on postoperative day 1, and CRP level in the first week postoperatively.

The following PPCs were recorded: pleural effusion, pulmonary thromboembolism, pneumo-mediastinum, pulmonary edoema, and transfusion-related lung injury (TRALI). All pulmonary complications that developed were consulted on by a pulmonologist and followed and treated in accordance with the patient’s clinic and current guidelines. Patient outcomes included time between debulking surgery and adjuvant therapy, need for intensive care and length of stay, length of postoperative hospital stay, readmission to hospital within 30 days, total length of hospital stay, mortality within 48 h of surgery, and mortality before discharge.

### Statistical Methods

In our study, SPSS 27.0 program (IBM Inc., Chicago, IL, USA) was used for a statistical analysis. Categorical data were expressed as percentage (%) and frequencies (N). The normal distribution assumptions of quantitative parameters were examined by considering histogram analyses, skewness, kurtosis, Kolomogorov–Smirnov analyses, and Q-q plot graphs. Quantitative data were expressed as median, minimum, and maximum (IQR: Interquartile range) or mean ± standard deviation. Comparisons between two independent groups were carried out with a Mann–Whitney U test or independent *t* test. Pearson chi-square or Fisher’s exact tests were used to compare categorical parameters, and a Bonferroni adjustment was performed when comparing column proportions. A logistic regression (LR) analysis was used for prognostic factors and effect states on binary conditions. In multivariate LR analyses, Box–Tidwell assumptions and model compatibility (goodness of fit) were checked with the Hosmer–Lemeshow test. The correlation relationships of quantitative parameters were evaluated by Pearson or Spearman correlation analyses. The cut-off values of quantitative parameters were detailed with an ROC analysis, and predictive capabilities were evaluated. A Type 1 error (α) was accepted as 5%, and the significance level (*p*) was accepted as <0.05. Preoperative imaging methods revealed pleural/pulmonary nodules in 11 patients, paracardiac lymph nodes in 25 patients, and cancer-related pleural ascites in 39 patients.

## 3. Results

**Figure 1:**
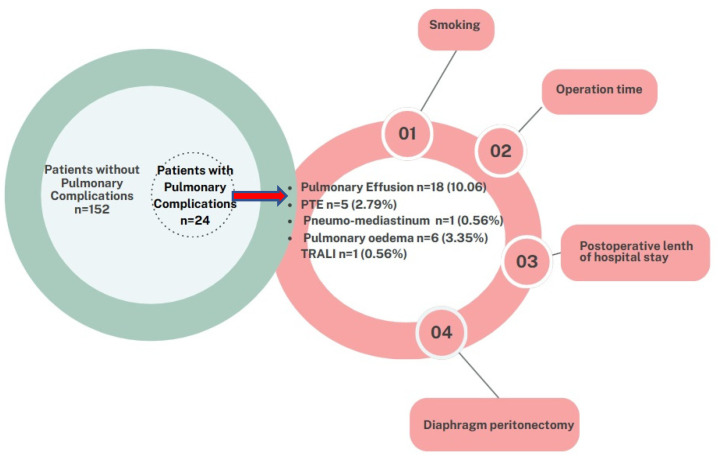
Pulmonary complications and statistically significant related factors.

A total of 176 patients with ovarian cancer underwent cytoreductive surgery for EOC during the study period. PPCS occurred in a total of 24 patients (13.4%). Of the complications observed, *n* = 18 (10.06%) were pulmonary effusion, *n* = 5 (2.79%) were pulmonary thromboembolism, *n* = 1 (0.56%) were pneumomediastinum, *n* = 6 (3.35%) were pulmonary oedema and *n* = 1 (0.56%) were TRALI (Figure 1). 

In addition, six patients in our study had cardiac complications, four of which were atrial fibrillation and two of which were sudden cardiac arrest. The clinicopathological factors in our study were as follows: mean age 57.8 ± 11.2 years, *n* = 40 (22.35%), ECOG performance status ≥ 2, smoking half pack/day *n* = 30 (18.4%), pack/day 1 (0.61%), major cardiac comorbidity *n* = 44 (24.58%), major pulmonary comorbidity *n* = 15 (8.38%), DM *n* = 30 (16.76%). Intraoperative observation revealed diaphragmatic disease in 49 patients, 37 of which were diffuse, and abdominal ascites were found in 31 patients with small volume and 35 patients with large volume. Maximum cytoreduction was 71.51% in 128 patients. A total of 116 (64.8%) patients underwent primary debulking and *n* = 63 (35.2%) patients underwent interval debulking, *n* = 98 (54.75%) patients underwent partial/total peritonectomy, *n* = 29 (16.2%) patients underwent diaphragmatic stripping, *n* = 23 (12.85%) patients underwent splenectomy and/or distal pancreatectomy, *n* = 120 (67.04%) patients underwent selective/systemic lymph node dissection. A total of 66 (36.87%) patients had stage III disease. The most common histotype was high-grade serous *n* = 122 (68.16%), followed by low-grade serous, clear-cell, low-grade endometrioid, mucinous, high-grade endometrioid, carcinosarcoma, squamous cell carcinoma derived from a mature teratoma, and the least number of Wolffian tumours. Intraoperative complications were seen in 18 patients, four of which were pleural openings during diaphragmatic stripping. *n* = 96 (53.63%) patients received intraoperative blood transfusions. Our mean operative time was 300 min. The mean preoperative Ca 125 level was 119, the albumin gr/dL was 3.9, the albumin gr/dL was 2.8 gr/dL on postoperative day 1, and the highest CRP level was 499 mg/dL on postoperative day 3. The mean interval between debulking surgery and adjuvant therapy was 36 (14–99) days, *n* = 107 (59.78%) patients required intensive care, and the median length of stay was 1 (0–69) day, while postoperative hospital stay was 16 (8–77) days. A total of 20 (11.56) patients were readmitted to hospital within 30 days, and mortality was observed in two patients within 48 h after surgery and in six patients before discharge (Table 1 and Table 2).

In a univariate analysis, serum albumin level before debulking surgery was 3.6 g/dL (*p* = 0.005), the postoperative day 1 albumin level was 2.6 g/dL (*p* = 0.003); statistically significant postoperative day 4–7 CRP levels, presence of diaphragmatic disease (*p* < 0.001), smoking (*p* = 0. 016), need for intraoperative blood transfusion (*p* = 0.024), type of cytoreduction (*p* = 0.011), peritonectomy (*p* < 0.001), diaphragm peritonectomy (*p* < 0.001), splenectomy/distal pancreatectomy (*p* = 0.018), and lymphadenectomy (*p* = 0.0039) were associated with PPCs. The pre-discharge mortality rate was 3 (12.5%) in the group with postoperative pulmonary complications and 3 (1.94%) in the group without (*p* = 0.016). (Table 3, Table 4 and Table 5).

For each parameter, a univariate logistic regression analysis was performed with respect to their effect profile on pulmonary complications, and their effects on pulmonary complications were examined. The analysis showed that increases in operative time (*p* < 0.001) and postoperative hospital stay (*p* = 0.002) were associated with pulmonary complications and had statistically significant effect profiles. PPC rates were found to be 3.79 and 3.19 times higher in patients with maximal and optimal cytoreduction, respectively. In addition, pulmonary complication rates were higher in patients who underwent peritonectomy (*p* = 0.001), diaphragmatic peritonectomy (*p* < 0.001), and splenectomy/pancreatectomy (*p* = 0.014) (11.43, 10.26, and 3.57 times higher, respectively). On the other hand, PPC rates were 4.56 times higher in patients who underwent systematic lymphadenectomy compared to those who did not (*p* < 0.05). The need for ICU follow-up was also associated with PPCs, and it was found that ICU follow-up status was associated with a 19.4 times higher risk of pulmonary complications (*p* = 0.004). Smoking increased the risk of PPCs by 2.93 times compared to non-smokers (*p* = 0.031). No significant effect profile was observed for other independent variables and other subgroups (*p* > 0.05) (Table 6).

Parameters that exhibited an effect profile on pulmonary complications (in univariate analysis) were included in the multivariate LR analysis, and parameters were adjusted for each other in the same model (as well as for age). After adjustment, smoking, operative time, length of postoperative hospital stay, and diaphragmatic peritonectomy were still statistically significant for pulmonary complications. The analysis showed that longer postoperative hospital stay and operation time were associated with pulmonary complications. In addition, the rate of pulmonary complications was 6.512 times higher in patients who underwent diaphragmatic peritonectomy (*p* = 0.014) and 26.1 times higher in smokers (*p* = 0.005). In the multivariate model, no significant effect profile was observed for other independent variables (Table 7).

**Figure 2:**
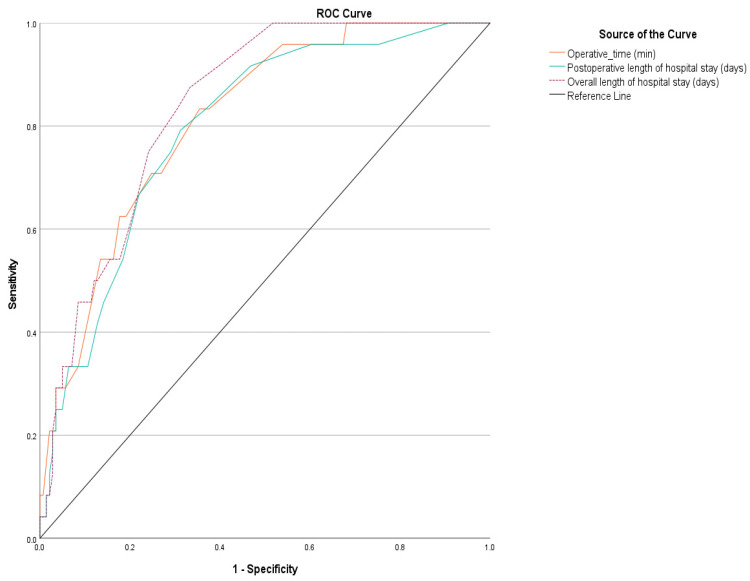
ROC analysis graphs for quantitative parameters in regard to pulmonary complications.

When an ROC analysis was performed for the quantitative parameters related to PPC, it was found that the sensitivity of the operation time was 83.3%, with a specificity of 64.5%, and the sensitivity of the postoperative hospital stay was 79.2%, with a specificity of 67.5 (*p* < 0.001, *p* < 0.001, *p* < 0.001) (Table 8, Figure 2).

## 4. Discussion

This study is one of the few articles to investigate factors associated with isolated PPCs following surgery for epithelial ovarian cancer. Our study results showed that PPCs were 6.512 times (*p* = 0.022) higher in patients who underwent diaphragmatic peritonectomy and 26.1 times (*p* = 0.005) higher in smokers. There was a statistically significant association between pulmonary complications and length of operation and postoperative hospital stay. In addition, the sensitivity and specificity of operation duration were 83.3% and 64.5%, and the sensitivity and specificity of postoperative hospital stay were 79.2% and 67.5% (*p* < 0.001, *p* < 0.001, *p* < 0.001, *p* < 0.001, *p* < 0.001).

In our study, the PPC rate in patients undergoing surgery for EOC was 13.4%. In the literature, studies have reported PPC rates ranging from 10% to 32.3% [7,11]. In our study, the most common complication was pulmonary effusion (*n* = 18, 10.06%), which is consistent with the literature [20,21]. Other complications included pulmonary oedema, pulmonary thromboembolism, pneumomediastinum, and TRALI.

In our study, the incidence of PPCs was 26.1 times higher in smokers than in non-smokers, a finding consistent with the literature [22,23,24,25,26]. A double-blind prospective study conducted at Mayo Hospital showed that those who had quit smoking for 8 weeks or more had a PPC rate of 12% compared with 33% in those who continued to smoke [27]. Encouraging patients to quit smoking, especially those scheduled for interval debulking, will significantly improve postoperative pulmonary complications by activating hepatic enzymes, strengthening the immune system, normalising carboxyhaemoglobin concentrations and increasing endo-bronchial ciliary function. Although it would be a mistake to consider smoking cessation as an “all or nothing” rule, primary debulking candidates who are unable to adhere to a smoking cessation programme of at least 4–6 weeks should still be encouraged to quit smoking [20,28,29].

Of the 616 patients diagnosed with epithelial ovarian cancer, 81 (13.2%) required diaphragmatic intervention. Moderate to severe pleural effusion was observed in 26 (32%) patients, with 16% undergoing pleural drainage and 11% undergoing placement of a single-lumen pleural catheter [30]. In a study of 150 patients with epithelial ovarian cancer, 96% of whom had stage IIIC disease, pleural effusion (33.3%), pneumonia (15.3%) and pneumothorax (7.3%) were the most commonly reported morbidities. The incidence of postoperative pleural effusion was 17.2 times (95% CI: 4.5–66.7), (*p* < 0.001) higher in stage IV disease, and full-thickness partial diaphragmatic resection was 4.9 times (95% CI: 1.2–19.9), (*p* = 0.028) higher [6]. In a study of 417 patients with epithelial ovarian cancer, 72 patients (17%) developed pulmonary complications. The occurrence of full-thickness diaphragm injury and/or diaphragm resection was found to increase the risk of pulmonary complications by a factor of 5.39 (95% CI 2.924–9.948, *p* < 0.001) and was also associated with a 2.285-fold decrease in overall survival (95% CI 1.232–4.241, *p* = 0.009) [31]. In line with the literature, this study found that the incidence of pulmonary complications was 4.9 times higher in patients who underwent partial and total diaphragmatic peritonectomy compared to other patients.

The results of our study showed a statistically significant relationship between operation time and postoperative hospital stay and the incidence of postoperative pulmonary complications. When evaluating the quantitative parameters related to pulmonary complications, operation time showed 83.3% sensitivity over 324 min, 79.2% sensitivity over 12.5 days postoperative hospital stay.

Although there was no significant result in multivariate analysis, preoperative albumin level below 3.6 g/dL and postoperative albumin level below 2.6 g/dL in univariate analysis may be a warning sign of postoperative pulmonary complications. In addition, we remind you that CRP levels, which are normal to rise to a certain level postoperatively, should be a warning sign of postoperative pulmonary complications, especially CRP levels of 260.4 mg/dL and above after 3 days. In the Hyperthermic Intraperitoneal Chemotherapy + Cytoreduction trial, which investigated the relationship between CRP levels and major postoperative complications in the literature, a level of 106 mg/dL and above on postoperative day 2 showed significance with an AUC of 0.658 (*p* = 0.006, 95% CI 0.56–0.76). The same study also analysed pre- and postoperative albumin levels but found no correlation [32].

The results of our study show that, although the postoperative hospital stay was significantly longer in the PPC group, there was no statistically significant difference in the time to start adjuvant chemotherapy between the two groups. This is a very important finding in terms of patient outcomes. This is probably due to the effective management of postoperative pulmonary complications, which did not adversely affect the time to start adjuvant therapy. The pre-discharge mortality rate was 3 (12.5%) in the group with postoperative pulmonary complications and 3 (1.94%) in the group without (*p* = 0.016).

Although a prospective data set was established, the limitation of our study is its retrospective nature. However, its strength is the detailed analysis of risk factors that may be associated with postoperative pulmonary complications and patient outcomes.

## 5. Conclusions

Patients with ovarian cancer make up a significant proportion of those diagnosed with gynaecological cancers. Treatment of this disease often requires extensive surgery, which can result in high rates of PPCs. A better understanding of the multifactorial aetiology of PPCs and the development of an appropriate perioperative management strategy may serve to reduce the adverse effects of these complications and thus contribute to improved patient outcomes. On the other hand, our results showed that some nominal variables, including the need for ICU follow-up, cytoreduction, peritonectomy, diaphragmatic peritonectomy, splenectomy/pancreatectomy, and lymphanedectomy, were not statistically significant after adjustment. This suggests that confounding factors such as age, smoking status, and other surgical concerns may have affected these factors, which should be taken into account in future studies.

## Figures and Tables

**Table 1:** Summary of overall distribution of quantitative parameters in the sample.

Parameter	Unit	Minimum	Maximum	Distribution ^†^
Age	years	22	82	57.8 ± 11.2
Seru Ca-125 level prior to DS (*n* = 169)	u/mL	3.4	19574.0	119 (3.4–19574)
Serum Albumin level prior to DS (*n* = 121)	g/dL	2.4	5.1	3.9 (2.4–5.1)
Postoperative Day 1 Albumin (*n* = 104)	g/dL	2.0	4.3	2.8 (2.0–4.3)
Day 1 CRP (*n* = 93)	mg/L	1.0	317.0	63 (1–317)
Day 2 CRP (*n* = 98)	mg/L	13	435	208.5 ± 91.3
Day 3 CRP (*n* = 98)	mg/L	30.3	499.0	219.1 ± 94.5
Day 4 CRP (*n* = 89)	mg/L	21.0	443.0	160 (21–443)
Day 5 CRP (*n* = 104)	mg/L	16.0	417.0	102 (16–417)
Day 6 CRP (*n* = 73)	mg/L	8.2	455.0	68 (8.2–455)
Day 7 CRP (*n* = 85)	mg/L	0.0	439.0	71 (0–439)
Operation time	min	120	610	300 (120–610)
Time interval between debulking surgery and adjuvant therapy	days	14	99	36 (14–99)
LOICU	days	0	69	1 (0–69)
Postoperative lenth of hospital stay	days	1	77	11 (1–77)
Overall lenth of hsopital stay	days	1	77	11 (1–77)

^†^ Parameters are expressed as IQR (Interquartile Range) [median, min and max] or mean ± SD. Abbrevations: DS: Debulking Surgery; CRP: C-reactive protein; LOICU: Length of intensive care unit.

**Table 2:** Summary of overall distributions of qualitative parameters.

Parameter	*n* (%)	Parameter	*n* (%)	Parameter	*n* (%)	Parameter	*n* (%)
**ECOG_PS**		**Pleural/pulmonary nodule**		**Tumor histotype**		**Pleural effusion**	
0	71 (39.66)	No	168 (93.85)	High grade serous	122 (68.16)	No	161 (89.94)
1	68 (37.99)	Yes	11 (6.15)	Wolffian adnexal	1 (0.56)	Yes	18 (10.06)
2	39 (21.79)	**PrO mediastinal/paracardic LN**		High grade endometrioid	4 (2.23)	**Pulmonary tromboembolism**	
3	1 (0.56)	No	154 (86.03)	Carcinosarcoma	3 (1.68)	No	174 (97.21)
**Major cardiac comorbiditiy**		Yes	25 (13.97)	Clear cell	11 (6.15)	Yes	5 (2.79)
No	135 (75.42)	**Debulking surgery**		Low grade serous	18 (10.06)	**Pneumo-mediastinum**	
Yes	44 (24.58)	Primary	116 (64.8)	Low grade (grade 1) endometroid	10 (5.59)	No	178 (99.44)
**Major pulmonary comorbidity**		Interval	63 (35.2)	Mucinous	7 (3.91)	Yes	1 (0.56)
No	164 (91.62)	**FIGO stage**		Seromucinous	1 (0.56)	**Pulmonary edema**	
Yes	15 (8.38)	I	34 (18.99)	Squamous cell	2 (1.12)	No	173 (96.65)
**DM**		II	17 (9.5)	**Postoperative chylous ascites**		Yes	6 (3.35)
No	149 (83.24)	III	66 (36.87)	No	166 (92.74)	**TRALI**	
Yes	30 (16.76)	IV	62 (34.64)	Yes	13 (7.26)	No	178 (99.44)
**Neoadjuvant chemotherapy**		**Cytoreduction**		**IO complication**		Yes	1 (0.56)
No	116 (64.8)	Maximal (no visible)	128 (71.51)	No	162 (97.01)	**Sudden cardiac arrest**	
3 cycles	25 (13.97)	Optimal (<1 cm)	38 (21.23)	Yes	5 (2.99)	No	177 (98.88)
4 cycles	25 (13.97)	Suboptimal (≥1 cm)	13 (7.26)	**IO need for blood transfusion**		Yes	2 (1.12)
≥6 cycles	13 (7.26)	**Peritonectomy (partial/total)**		No	83 (46.37)	**Atrial fibrillation**	
**Ascites**		No	81 (45.25)	Yes	96 (53.63)	No	175 (97.77)
No	113 (63.13)	Yes	98 (54.75)	**Need for ICU**		Yes	4 (2.23)
Small volumes	31 (17.32)	**Diaphragm peritonectomy**		No	72 (40.22)	**Smoking history**	
Large volumes	35 (19.55)	No	150 (83.8)	Yes	107 (59.78)	No	132 (80.98)
**Diaphragmatic disease**		Yes	29 (16.2)	**30-day hospital readmission**		Half pack/daily	30 (18.4)
No	130 (72.63)	**Splenectomy/pancreatectomy**		No	153 (88.44)	1 pack/Daily	1 (0.61)
Localized foci	12 (6.7)	No	156 (87.15)	Yes	20 (11.56)	**Cardiac complication (overall)**	
Diffuse military	37 (20.67)	Yes	23 (12.85)	**Mortality—48h after surgery**		No	173 (96.6)
**Preoperative pleural effusion**		**Lymphadenectomy**		No	177 (98.88)	Yes	6 (3.4)
No	140 (78.21)	No	59 (32.96)	Yes	2 (1.12)	**Pulmonary complication (overall)**	
Yes	39 (21.79)	Selective LN debulking	5 (2.79)	**Mortality prior to discharge**		No	155 (86.6)
		Systematic pelvic-paraaortic	115 (64.25)	No	173 (96.65)	Yes	24 (13.4)
				Yes	6 (3.35)		

Abbrevations: ICU: Intensive care unit; IO: Intraoperative; PrO: Pre-operative; TRALI: Transfusion related acute lung injury; LN: Lymph node; DM: Diabetes mellitus.

**Table 3:** Comparison of quantitative parameters according to pulmonary complication status.

		Pulmonary Complications		*p*-Value
		No (*n* = 155, 86.6%)	Yes (*n* = 24, 13.4%)	
Parameter	Unit	Distribution *		
Age	years	58.0 ± 11.0	59.0 ± 10.0	0.480 ^a^
Seru Ca-125 level prior to DS	u/mL	113 (3.4–13032)	198.5 (14–19574)	0.374 ^b^
Serum Albumin level prior to DS	g/dL	4 (2.4–5.1)	3.6 (2.8–4.4)	**0.005** ^b^
Postoperative day 1 Albumin	g/dL	2.9 (2–4.3)	2.6 (2–3.2)	**0.003** ^b^
Day 1 CRP	mg/L	59.3 (1–306)	104.3 (4–317)	0.406 ^b^
Day 2 CRP	mg/L	205.4 ± 85	225.1 ± 120.6	0.432 ^a^
Day 3 CRP	mg/L	210 ± 98.4	260.4 ± 60.8	**0.008** ^a^
Day 4 CRP	mg/L	145 (21–442)	268.3 (82–443)	**<0.001** ^b^
Day 5 CRP	mg/L	91.5 (16–417)	195 (38–412)	**<0.001** ^b^
Day 6 CRP	mg/L	57.3 (8.2–455)	172 (26–307)	**0.003** ^b^
Day 7 CRP	mg/L	58.5 (0–439)	157 (21–319)	**<0.001** ^b^
Operation time	min	300 (120–570)	420 (255–610)	**<0.001** ^b^
Time interval between DS and adjuvant therapy	days	36 (14–99)	34 (22–73)	0.646 ^b^
LOICU	days	1 (0–69)	2 (1–38)	**<0.001** ^b^
Postoperative lenth of hospital stay	days	10 (1–69)	16 (8–77)	**<0.001** ^b^
Overall lenth of hospital stay	days	11 (1–69)	19 (11–77)	**<0.001** ^b^

Abbrevations: DS: debulking surgery; LOICU: Lenth of stay in intensive care unit; CRP: C-reactive protein; * Fisher’s exact test; ^a^ Independent *t* test; ^b^ Mann-whitney U test.

**Table 4:** Comparison of quantitative parameters according to pulmonary complication status.

		Pulmonary Complications		*p*-Value
		No (*n* = 155, 86.6%)	Yes (*n* = 24, 13.4%)	
Parameter		Distribution *		
ECOG_PS	0	66 (42.58%)	5 (20.83%)	0.151 *
	1	57 (36.77%)	11 (45.83%)	
	2	31 (20%)	8 (33.33%)	
	3	1 (0.65%)	0 (0%)	
Major cardiac comorbiditiy	No	118 (76.13%)	17 (70.83%)	0.575 **
	Yes	37 (23.87%)	7 (29.17%)	
Major pulmon. comorbiditiy	No	141 (90.97%)	23 (95.83%)	0.697 *
	Yes	14 (9.03%)	1 (4.17%)	
DM	No	131 (84.52%)	18 (75%)	0.247 *
	Yes	24 (15.48%)	6 (25%)	
Neoadjuvant chemotherapy	No	100 (64.52%)	16 (66.67%)	0.204 *
	3 cycles	23 (14.84%)	2 (8.33%)	
	4 cycles	19 (12.26%)	6 (25%)	
	≥6 cycles	13 (8.39%)	0 (0%)	
Ascites	No	104 (67.1%) ^a^	9 (37.5%) ^b^	**0.008** *
	Small volumes	26 (16.77%)	5 (20.83%)	
	Large volumes	25 (16.13%) ^a^	10 (41.67%) ^b^	
Diaphragmatic disease	No	121 (78.06%) ^a^	9 (37.5%) ^b^	**<0.001** *
	Localized foci	9 (5.81%)	3 (12.5%)	
	Diffuse military	25 (16.13%) ^a^	12 (50%) ^b^	
Preoperative pleural effusion	No	123 (79.35%)	17 (70.83%)	0.347 **
	Yes	32 (20.65%)	7 (29.17%)	
Pleural/pulmonary nodule	No	145 (93.55%)	23 (95.83%)	0.999 *
	Yes	10 (6.45%)	1 (4.17%)	
PrO mediastinal/paracardic LN	No	134 (86.45%)	20 (83.33%)	0.751 *
	Yes	21 (13.55%)	4 (16.67%)	
FIGO stage	I	32 (20.65%)	2 (8.33%)	0.066 *
	II	17 (10.97%)	0 (0%)	
	III	57 (36.77%)	9 (37.5%)	
	IV	49 (31.61%)	13 (54.17%)	
Tumor histotype	High grade serous	106 (68.39%)	16 (66.67%)	0.910 *
	Wolffian adnexal	1 (0.65%)	0 (0%)	
	High grade endometrioid	4 (2.58%)	0 (0%)	
	Carcinosarcoma	2 (1.29%)	1 (4.17%)	
	Clear cell	9 (5.81%)	2 (8.33%)	
	Low grade serous	15 (9.68%)	3 (12.5%)	
	Low grade (grade 1) endometrioid	9 (5.81%)	1 (4.17%)	
	Mucinous	6 (3.87%)	1 (4.17%)	
	Seromucinous	1 (0.65%)	0 (0%)	
	Squamous cell	2 (1.29%)	0 (0%)	
Postoperative chylous ascites	No	142 (91.61%)	24 (100%)	0.221 *
	Yes	13 (8.39%)	0 (0%)	
Intraoperative complication	No	141 (96.58%)	21 (100%)	0.999 *
	Yes	5 (3.42%)	0 (0%)	
Intraoperative need for blood transfusion	No	77 (49.68%)	6 (25%)	**0.024** **
	Yes	78 (50.32%)	18 (75%)	
Need for ICU	No	71 (45.81%)	1 (4.17%)	**<0.001** **
	Yes	84 (54.19%)	23 (95.83%)	
30-day hospital readmission	No	137 (90.13%)	16 (76.19%)	0.073 *
	Yes	15 (9.87%)	5 (23.81%)	
Mortality—48h after surgery	No	153 (98.71%)	24 (100%)	0.999 *
	Yes	2 (1.29%)	0 (0%)	
Mortality prior to discharge	No	152 (98.06%)	21 (87.5%)	**0.033** *
	Yes	3 (1.94%)	3 (12.5%)	
Smoking	No	118 (83.68%) ^a^	14 (63.64%) ^b^	**0.016**
	Half pack/daily	23 (16.31%)	7 (31.82%)	
	1 pack/daily	0 (0%) ^a^	1 (4.54%) ^b^	

Abbrevations: ECOG-PS: Eastern Cooperative Oncology Group Performance Status; DS: debulking surgery; LOICU: Lenth of stay in intensive care unit; PrO: Preoperatif; * Fisher’s exact test; ** Pearson chi-square analysis. Statistically significant differences for column proportions are marked with the letters (a) and (b).

**Table 5:** Evaluation of the distributional relationships between surgical procedures and pulmonary complications.

		Pulmonary Complications		*p*-Value
		No (*n* = 155, 86.6%)	Yes (*n* = 24, 13.4%)	
Surgery Type	Procedure Status	Distribution *		
Debulking surgery	Primary	100 (64.52%)	16 (66.67%)	0.837 *
	Interval	55 (35.48%)	8 (33.33%)	
Cytoreduction	Maximal (no visible)	117 (75.48%) ^a^	11 (45.83%) ^b^	**0.011** *
	Optimal (<1 cm)	28 (18.06%) ^a^	10 (41.67%) ^b^	
	Suboptimal (≥1 cm)	10 (6.45%)	3 (12.5%)	
Peritonectomy (partial/total)	No	79 (50.97%)	2 (8.33%)	**<0.001** *
	Yes	76 (49.03%)	22 (91.67%)	
Diaphragm peritonectomy	No	139 (89.68%)	11 (45.83%)	**<0.001** **
	Yes	16 (10.32%)	13 (54.17%)	
Splenectomy/pancreatectomy	No	139 (89.68%)	17 (70.83%)	**0.018** **
	Yes	16 (10.32%)	7 (29.17%)	
Lymphadenectomy	No	44 (28.39%) ^a^	15 (62.5%) ^b^	**0.003** **
	Selective LN debulking	4 (2.58%)	1 (4.17%)	
	Systematic pelvic-paraaortic	107 (69.03%) ^a^	8 (33.33%) ^b^	

Abbrevations: DS: debulking surgery; LOICU: Lenth of stay in intensive care unit; * Pearson chi-square analysis; ** Fisher’s exact test. Statistically significant differences for column proportions are marked with the letters (a) and (b).

**Table 6:** Univariate Logistic regression analysis of parameters in predicting pulmonary complications.

Logistic Regression (Pulmonary Complication)						
Parameters	B	-2LL	Nagelkerke R^2^	*p*-Value	Exp(B)	95% CI
Age	0.014	140.566	0.005	0.478	1.014	0.976–1.054
Smoking (+) ^†^	1.076	124.688	0.048	**0.031**	2.932	1.104–7.786
Time interval between debulking surgery and adjuvant therapy	−0.004	108.203	0.001	0.845	0.996	0.960–1.034
Operation time	0.012	110.731	0.260	**<0.001**	1.012	1.007–1.017
Lenth of stay in ICU	0.036	110.060	0.026	0.205	1.037	0.980–1.097
Postoperative lenth of hospital stay	0.078	127.660	0.130	**0.002**	1.081	1.028–1.137
Overall lenth of hospital stay	0.091	122.771	0.178	**<0.001**	1.096	1.043–1.151
Need for ICU follow-up (+)	2.967	121.915	0.186	**0.004**	19.440	2.561–147.563
30-day hospital readmission	−1.049	125.010	0.032	0.071	0.350	0.112–1.092
Debulking surgery	−0.095	141.033	0.0004	0.837	0.909	0.366–2.259
Cytoreduction (+) ^a^	0.781	134.985	0.061	**0.011**	2.183	1.198–3.978
Peritonectomy (partial/total) (+)	2.437	123.132	0.175	**0.001**	11.434	2.599–50.3
Diaphragm peritonectomy (+)	2.329	118.545	0.217	**<0.001**	10.267	3.950–26.68
Splenectomy/pancreatectomy (+)	1.275	135.709	0.054	**0.014**	3.577	1.289–9.930
Lymphadenectomy (+) ^b^	−0.755	130.123	0.109	**0.001**	0.470	0.297–0.745

Abbrevations: ICU: Intensive Care Unit. ^a^ Subgroup comparison with the reference category: Optimal (OR) = 3.79 (*p* < 0.05); Suboptimal (OR) = 3.19 (*p* < 0.05). ^b^ Subgroup comparison with the reference category: Selective LN debulking (OR) = 4.56 (*p* < 0.05). ^†^ Binary grouped as smokers and non-smokers.

**Table 7:** Multivariate Logistic regression analysis of parameters in predicting pulmonary complications.

Logistic Regression (Pulmonary Complication)				
-2LL = 79.711; Nagelkerke R^2^ = 0.519				
Parameters	B	*p*-Value	Exp(B)	95% CI
Age	0.063	0.154	1.065	0.977–1.162
Smoking (+) ^†^	3.266	**0.005**	26.198	2.666–257.398
Operation time	0.011	**0.014**	1.011	1.002–1.019
Postoperative lenth of hospital stay	0.194	**0.037**	0.823	0.686–0.988
Overall lenth of hospital stay	0.259	**0.008**	1.296	1.070–1.570
Need for ICU (+)	0.693	0.575	1.999	0.178–22.508
Cytoreduction (+)	0.552	0.355	1.737	0.539–5.592
Peritonectomy (partial/total) (+)	1.186	0.317	3.273	0.321–33.394
Diaphragm peritonectomy (+)	1.874	**0.022**	6.512	1.309–32.396
Splenectomy/pancreatectomy (+)	−0.254	0.780	0.775	0.130–4.631
Lymphadenectomy (+)	−0.754	0.097	0.471	0.193–1.146

Abbrevations: ICU: Intensive Care Unit. Box-tidwell assumption for quantitative parameters were met. Model compatibility (goodness of fit) was checked and confirmed by Hosmer and Lemeshow test (*p* = 0.378). ^†^ Binary grouped as smokers and non-smokers.

**Table 8:** ROC analysis for quantitative parameters in regard to pulmonary complications and cut-off values.

	AUC (%95 CI)	Cut-off ^†^	*p*-Value	Sensitivity (%)	Specificity (%)
Operation time (min)	0.813 (0.731–0.894)	324.5	**<0.001**	83.3%	64.5%
Postoperative lenth of hospital stay (days)	0.791 (0.702–0.880)	12.5	**<0.001**	79.2%	67.5%
Overall lenth of hospital stay (days)	0.835 (0.767–0.904)	12.5	**<0.001**	87.5%	65.8%

Abbrevations: AUC: Area under curve (eğri altında kalan alan), ROC: Receiver operating characteristic, CI: Confidence Interval (Güven Aralığı) Referans Kategori: Kontrol Grubu. † Youden J index was used in determining the cut-off values.

## Data Availability

The data generated in the present study may be requested from the corresponding author.

## Abbreviations

ECOG	Eastern Cooperative Oncology Group
EOC	Epithelial ovarian cancer
DM	Diabetes mellitus
PPCs	Postoperative pulmonary complications
TRALI	Transfusion-related lung injury
